# Human gingival fibroblast response to enamel matrix derivative, porcine recombinant 21.3-kDa amelogenin and 5.3-kDa tyrosine-rich amelogenin peptide

**DOI:** 10.1007/s13577-017-0164-z

**Published:** 2017-05-03

**Authors:** Marzena Wyganowska-Swiatkowska, Paulina Urbaniak, Daniel Lipinski, Marlena Szalata, Malgorzata Kotwicka

**Affiliations:** 1 Department of Conservative Dentistry and Periodontology, University of Medical Sciences, Bukowska 70, 60-812 Poznan, Poland; 2 Department of Cell Biology, University of Medical Sciences, Rokietnicka 5D, 60-806 Poznan, Poland; 3 Department of Biochemistry and Biotechnology, Poznan University of Life Sciences, Dojazd 11, 60-632 Poznan, Poland

**Keywords:** Gingival fibroblast, Enamel matrix derivative, Amelogenin, TRAP, Proliferation, Migration, Cell cycle

## Abstract

Enamel matrix derivative (EMD) containing a variety of protein fractions has been used for periodontal tissue regeneration. It is suggested that the proteins contained in EMD positively influence gingival fibroblasts migration and proliferation. Effects of EMD as well as of porcine recombinated 21.3-kDa amelogenin (prAMEL) and 5.3-kDa tyrosine-rich amelogenin peptide (prTRAP) on human gingival fibroblast (HGF-1, ATCC; USA) cell line were investigated. Real-time cell analysis (xCELLigence system; Roche Applied Science) was performed to determine the effects of EMD, prAMEL and prTRAP (12.5–50 μg/mL) on HGF-1 cell proliferation and migration. The effect of treatment on cell cycle was determined using flow cytometry. EMD significantly increased HGF-1 cell proliferation after 24- and 48-h incubation. Individually, prAMEL and prTRAP also increased HGF-1 cell proliferation; however, the difference was significant only for prAMEL 50 µg/mL. prAMEL and TRAP significantly increased HGF-1 cell migration after 60- and 72-h incubation. Cell cycle analysis showed significant decrease of the percentage of cells in the G0/G1 phase and a buildup of cells in the S and M phase observed after EMD and prAMEL stimulation. This process was ligand and concentration-dependent. The various molecular components in the enamel matrix derivative might contribute to the reported effects on gingival tissue regeneration; however, biologic effects of prAMEL and prTRAP individually were different from that of EMD.

## Introduction

Periodontium is a dynamic structure consistent of epithelial tissue and different type of connective tissue, which have complex interrelationship. This interaction between epithelial and connective tissue, stimulated by enamel matrix proteins (EMP) during dentogenesis is mimicked by enamel matrix derivative (EMD), over healing and regeneration process. The beneficial outcome of EMD on periodontal regeneration has been proven through clinical and in vitro data. EMD in the treatment of gingival recession also improves clinical parameters, suggesting its beneficial influence on gingival fibroblasts (GF) [1]. Fibroblasts are a heterogeneous group of cells with distinct properties and functions, responsible for regulation of tissue development, organogenesis, and homeostasis [2, 3]. GF are considered as a potential source of pluripotent cells for regeneration not only of periodontal, but also out-oral cavity structure [4]. They influence keratinocyte migration and play an important role in oral wound healing, a complex process which includes cell migration, cell attachment to various extracellular matrix components, and cell proliferation [5]. Interestingly, fibroblast phenotypes are modulated by other factors, including developmental origin or the local tissue niche [6]. The genetic origins define theirs different tissue-specific “memory” and interaction with other cells [7], as well as response for stimulation of the same factor. It also determines the outcome of periodontal wound healing—repair or regeneration [8]. Although GF are phenotypically and functionally different from skin fibroblasts [9] and periodontal fibroblasts [3] in all type of cells the reaction on EMD was observed. EMD, amelogenin—its main component, amelogenin degradation products (TRAP) or alternatively spliced products (LRAP) have distinct biochemical properties [10, 11]. It was indicated that EMD significantly increased GF proliferation [12, 13], and this process was dose and time related. Amelogenin influences gingival fibroblasts adhesion rather than proliferation. Moreover, the inhibiting effect of recombinant amelogenin on GF migration is suggested [14], as well as TRAP biological inactivity [15]. Because of different GF reactions on whole EMD and their components, and the possible gain in gingival recession treatment after using the separate stimulation, we decided to analyse the influence of various EMD, AMEL and TRAP concentrations on human gingival fibroblast in the condition most similar to in vivo. xCELLigence real-time cell analysis (RTCA) system as a non-invasive and label-free approach to assess cell proliferation in real-time on a cell culture level was used.

## Materials and methods

### Proteins

Lyophilized EMD were obtained from Institut Straumann AG, Switzerland, and prepared according to Institute Straumann operating protocols to the working solution 12.5, 25 and 50 µg/mL. Porcine recombinant 21.3-kDa amelogenin (prAMEL) and porcine recombinant 5.3-kDa tyrosine-rich amelogenin peptide TRAP were synthesized.

### Amelogenin synthesis

Porcine recombinant AMEL protein (prAMEL) was synthesized by BLIRT S.A. (Gdańsk, Poland). The protein sequence of *Sus scrofa* AMEL was obtained from the UniProt database (http://www.uniprot.org/, accession no. Q861X0). This sequence, with an added glutathione *S*-transferase (GST) tag to increase protein solubility, was the following: ENFLYQGSMPLPPHPGHPGYINFSYEDLYLEAIRIDRTAFVLTPLKWYQNMIRHPYTSYGYEPMGGWLHHQIIPVVSQQTPQSHALQPHHHIPMVPAQQPGIPQQPMMPLPGQHSMTPTQHHQPNLPLPAQQPFQPQPVQPQPHQPLQPQSPMHPIQPLLPQPPLPPMFSMQSLLPDLPLEAWPAT. The resulting prAMEL with GST was approximately 49 kDa.

### TRAP synthesis

The construct contains TRAP fragment of porcine amelogenin gene under the control of T7 promoter in expression vector pET-22b(+). The 5′ end of the TRAP was modified by addition ATG codon, sequence encoding 6 histidine residues and the enterokinase recognition site. General procedures for manipulating DNA were carried out according to Sambrook and Russel [16]. PCR reagent, restriction enzymes and T4DNA ligase were purchased from Sigma, Fermentas or New England Biolabs. The whole construct was sequenced using automated genetic analyzers (Applied Biosystems Prism). *E. coli* Rosetta 2(DE3) pLysS strains [genotype: F^−^ *ompT hsdS*_B_(r_B_^−^ m_B_^−^) *gal dcm* (DE3) pLysSRARE2 (Cam^R^)] (Novagen) as host for gene expression experiments was grown in LB medium supplemented with ampicillin (100 µg/mL) and chloramphenicol (34 µg/mL).

Both amelogenin and TRAP synthesis was described in details in our previous study [39].

### Cell culture

All experiments were conducted on human gingival fibroblast cell line (HGF-1 ATCC^®^ CRL-2014, American Type Culture Collection; USA). HGF-1 cell line was transferred in aseptic conditions from freezing medium DMEM/F12 (1:1) (Gibco; USA), 10% foetal bovine serum (FBS; Gibco), 10% DMSO (Gibco), to 90-mm sterile petri dish (Sarstedt, Germany) containing 10 mL of growth medium with the following composition: DMEM/F12 (1:1) medium, 10% FBS, antibiotics: penicillin 100 μg/mL and streptomycin 100 μg/mL (Gibco) and 2 mmol/L l-glutamine (Gibco). Cells were grown in 37 °C, 5% CO_2_ and 95% humidity conditions. Cells were cultured until 90% confluence, washed with phosphate buffered saline (PBS) and trypsinized (0.25% trypsin containing 0.01% EDTA). After 5 min of incubation, complete growth medium was added, and cell suspension was transferred to petri dishes. The culture medium was added at the volume ratio of 1/10.

### Cell proliferation and monitoring

Cell proliferation was monitored in real-time using the xCELLigence system E-Plate. The electronic impedance of the sensor electrodes was measured to allow monitoring and detection of physiologic changes of the cells on the electrodes. The voltage applied to the electrodes during real-time cell analyser measurement was about 20 mV root mean square. The impedance measured between electrodes in a well depends on electrode geometry, ion concentration in the well, and if cells are attached to the electrodes. In the presence of cells, cells attached to the electrode sensor surfaces act as insulators and thereby alter the local ion environment at the electrode–solution interface, leading to increased impedance. Thus, more cells are growing on the electrodes, increasing the value of electrode impedance. The electrical impedance value of each well was automatically monitored by xCELLigence system and expressed as a cell index (CI) value. Each experiment was performed five times. The external control plate contained cells non-stimulated with the proteins.

During the proliferation measurements after reaching confluence cells were passaged with 0.25% trypsin. After seeding 200 μL of cell suspensions into the wells (10,000 cells/well) of the E-plate 96, HGF-1 cells were incubated in order to obtain cell index value equal about 1. Afterwards cells were treated with 12.5, 25 and 50 µg/mL dilutions of EMD, prAMEL and prTRAP and released by the metallic alloy material and monitored every 15 min for 72 h. The control plate contained non-stimulated cells. The evaluation was performed 12, 24, and 48 h after stimulation.

### Monitoring cell migration

The rate of cell migration was monitored in real-time with the xCELLigence system (CIM-pates). The cells were passaged and placed on upper chamber of CIM-plate 16 in FBS-free medium. The lower chamber of CIM-plate 16 contained 160 μL of medium with 10% of FBS, as an attractant. Electrodes located between lower and upper chamber measured cell migration. Right after seeding 200 μL of the cell suspensions into the wells (20,000 cells/well), HGF cells were treated with EMD, prAMEL and prTRAP and monitored every 15 min for 72 h. The control plate contained cells non-stimulated with the proteins.

### Cell cycle analysis

The cells were seeded in 60-mm culture dishes at a density of 5 × 105 cells/dish and allowed to adhere overnight. Following 15 min of incubation with EMD, prAMEL or prTRAP, the cells were washed twice with PBS and the solutions were then replaced with regular growth medium, and the cells were grown under standard conditions for 48 h. Subsequently, the cells were trypsinized (trypsin; Cytogen) and fixed with ice-cold 70% ethanol at −20 °C for 24 h. Subsequently, the cells were centrifuged, washed once with PBS, and then incubated with RNAse A (50 μg/mL in PBS) for 30 min. After centrifugation, the supernatant with RNAse A was removed and intracellular DNA was labelled with 0.5 mL of cold propidium iodide (PI) solution (0.1% Triton X-100, 0.1 mM EDTA, 50 μg/mL PI in PBS) on ice for 30 min in the dark. Cell cycle distribution was measured using a FACSCalibur flow cytometer (BD Biosciences, San Jose, CA, USA). For each experiment, 10,000 cells were examined. The fluorescence of PI was excited using an argon laser (488 nm). The emission of red fluorescence of PI was detected in the FL3 channel (>650 nm) All data were collected and analysed using CellQuest Pro software (v.5.2.1) (Becton-Dickinson, Franklin Lakes, NJ, USA). The distribution of cells in the cell cycle (G0/G1, S and G2/M) and apoptosis were calculated using the ModFit LT program for cell cycle analysis (Verity Software House Inc., Topsham, ME, USA).

### Statistical analysis

The Shapiro–Wilk test was used as the normality test of continuous variables. Homogeneity of variance was assessed with Levene test. A one-way ANOVA, followed by Tukey (RIR) post hoc test were used to analyse the relationship between various proteins. Repeated measures one-way ANOVA with Tukey–Kramer multiple comparisons test were used to evaluate changes in time for particular ligands. The analysis was made using Statistica 10 software (StatSoft Inc., Tulsa, OK). Data were presented as mean ± standard deviation and considered statistically significant at *P* < 0.05.

## Results

### Proteins synthesis

**Fig. 1:**
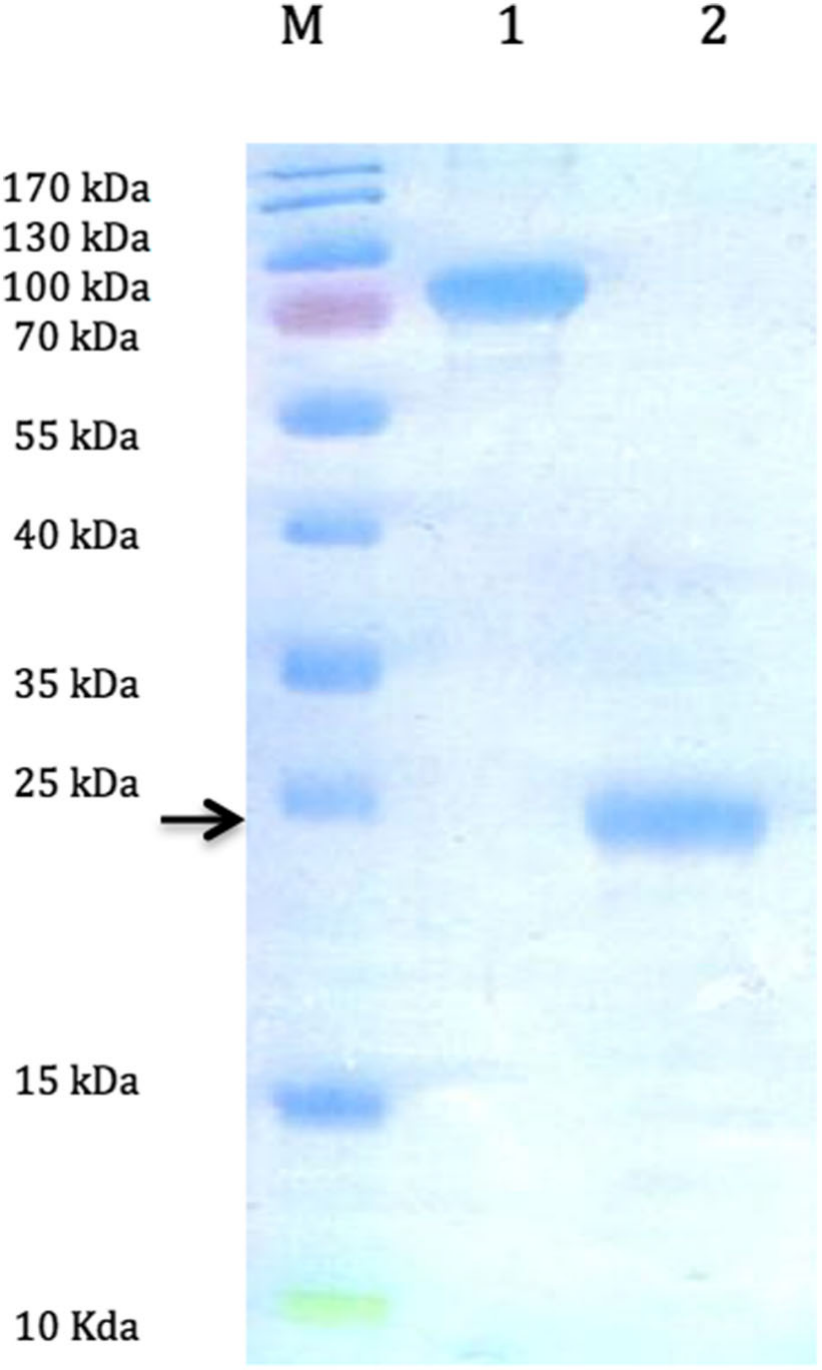
An electropherogram of separation of proteins in 12% polyacrylamide gel. Proteins were stained with Coomassie blue. Evaluation of amelogenin purification process. *Lane M* Thermo Scientific™ PageRuler™ Prestained Protein Ladder, 10–170 kDa; *lane 1* fusion protein amelogenin—glutathione *S*-transferase (GST) (AMEL-GST, 49 kDa); *lane 2* amelogenin after removal of glutathione *S*-transferase tag (21.3 kDa)

**Fig. 2:**
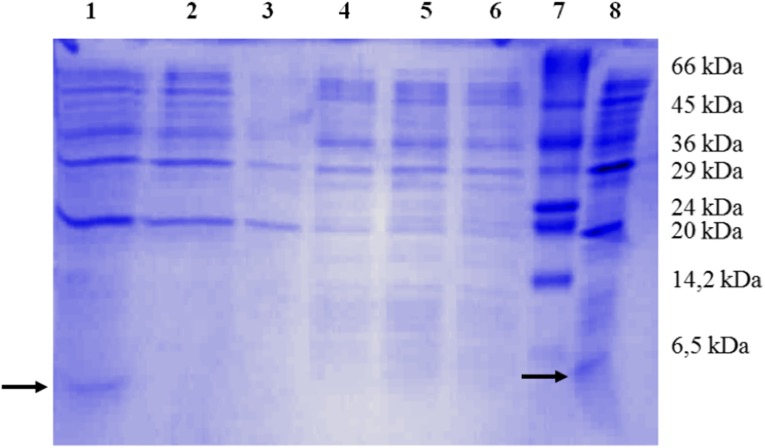
TRAP synthesis. 17% (w/v) SDS-PAGE analysis of the whole cell lysate samples from overexpressed cell cultures. Proteins were stained with Coomassie Brilliant Blue. *Lane 1* bacterial sediment after 9 h culture with IPTG at 37 °C; *lane 2* bacterial sediment after 16-h culture with IPTG at 37 °C; *lane 3* bacterial sediment from non-recombinant bacteria after 16-h culture with IPTG at 37 °C, negative control; *lane 4* bacterial sediment after 9-h culture with IPTG at 4 °C; *lane 5* bacterial sediment after 16-h culture with IPTG at 4 °C; *lane 6* bacterial sediment from non-recombinant bacteria after 16-h culture with IPTG at 4 °C, negative control; *lane 7* molecular mass markers (SigmaMarker low molecular weight range 6.5–66 kDa); *lane 8* culture medium after 16-h culture with IPTG at 37 °C. *Arrows* indicate recombinant peptide

Recombinant Amelogenin and TRAP synthesis was performed using IPTG induction of overexpression at 37 °C as it was described earlier [39]. Purification of each fragment needed evaluation of the growth condition. According to presence of different protein Tag, glutathione *S*-transferase (GST) tag for Amelogenin and Histidine tag for TRAP, typical purification methods dedicated for each tag were performed [39]. Electrophoretic image of purification of Amelogenin is shown in Fig. 1 and evaluation of overexpression condition of TRAP fragment in Fig. 2.

### Cell proliferation

**Fig. 3:**
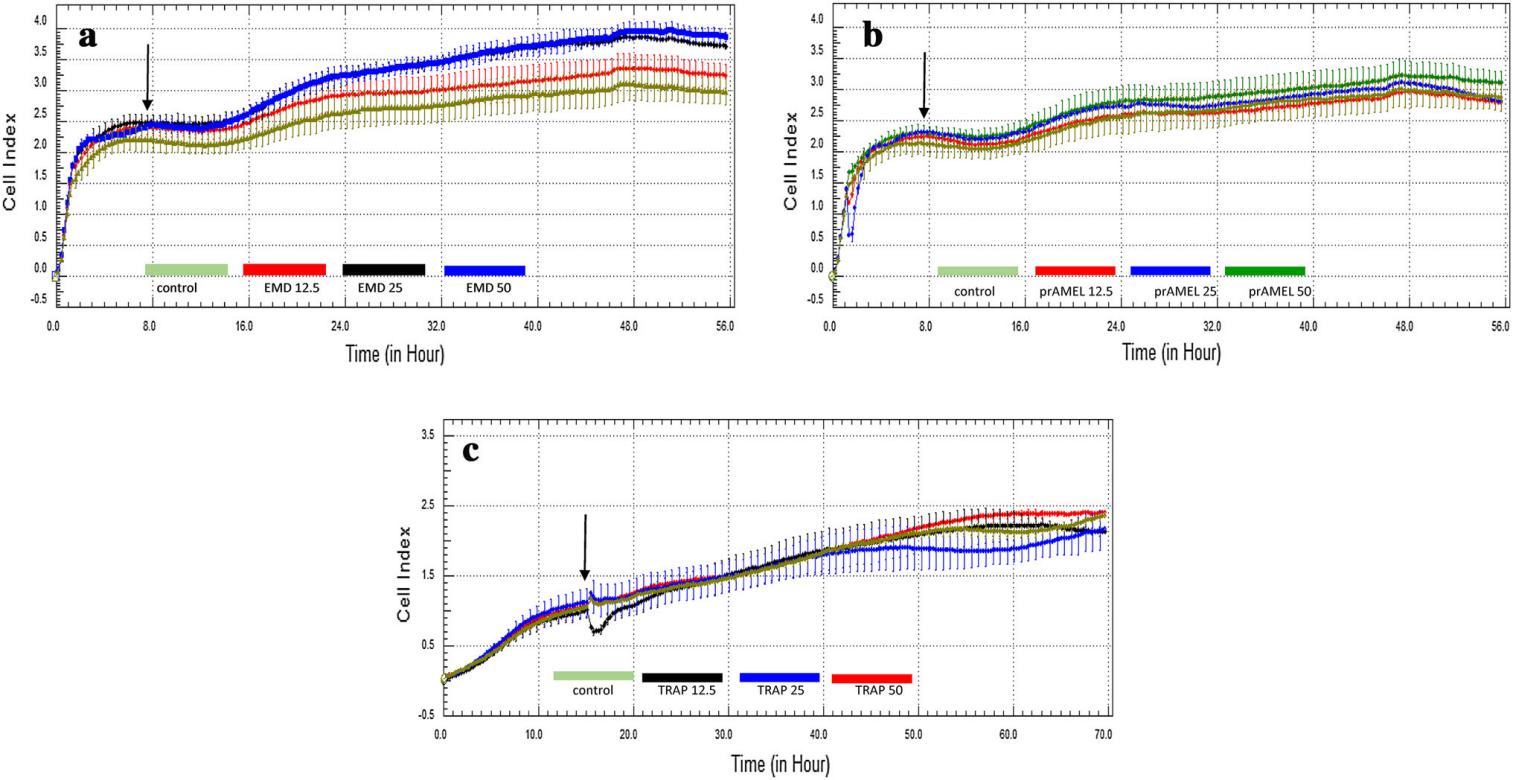
Effect of EMD on the rate of proliferation of HGF-1 cells. HGF-1 cells were incubated with EMD (**a**), prAMEL (**b**) or TRAP (**c**) for 48 h. The rate of proliferation was monitored in real-time using the xCELLigence system; *arrows* indicate the moment of ligand application; representative graph

**Fig. 4:**
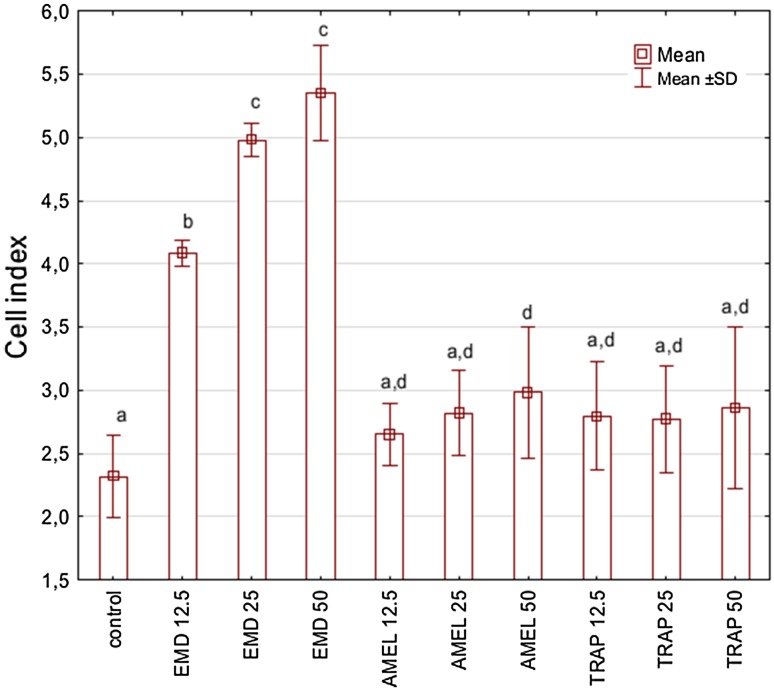
Effect of EMD, prAMEL and TRAP on the rate of proliferation of HGF-1 cells 48 h after stimulation. Data obtained from 5 separate analyses are expressed as mean ± standard deviation. *Different subscript letters above each bar* denote significant difference, *P* < 0.05 [one-way ANOVA with the Tukey (RIR) post hoc test]

Cell behaviour was monitored using RTCA over a period of 48 h after EMD, prAMEL or prTRAP stimulation. Representative graph comparing the rate of CI in stimulated HGF-1 cells is shown in Fig. 3a–c. No significant differences of the rate of proliferation among all analysed groups were observed after 12-h incubation (Table 1). RTCA analysis performed after 24- or 48-h incubation showed a significant increase of CI in EMD-stimulated cell, for all applied concentrations compared to both the controls and to the prAMEL and prTRAP (Table 1; Fig. 4). The significant difference concerned the 12.5 µg/mL EMD versus 25 µg/mL EMD and 12.5 µg/mL EMD versus 50 µg/mL EMD, but not 25 µg/mL EMD versus 50 µg/mL EMD. Moreover, all doses of prAMEL and prTRAP administered for 48 h cause an increase in proliferation rate in comparison to control cells, but the difference was significant only for prAMEL 50 µg/mL (*P* < 0.05; Fig. 4).

**Table 1:** Effect of enamel matrix proteins on the rate of proliferation of HGF-1 cells

EMP added, µg/mL	Cell index value, mean ± standard deviation			
	Time of incubation			
	12 h	24 h	48 h	*P* value
Control	2.1 ± 1.2	2.3 ± 1.2	2.6 ± 0.3	<0.05
EMD, 12.5	3.5 ± 0.2	4.0 ± 0.5	4.1 ± 0.1	<0.05
EMD, 25.0	3.3 ± 0.5	3.7 ± 0.4	4.9 ± 0.1	<0.01
EMD, 50.0	4.0 ± 0.3	4.3 ± 0.3	5.3 ± 0.3	<0.001
prAMEL, 12.5	2.4 ± 1.2	2.8 ± 1.1	2.6 ± 0.2	<0.05
prAMEL, 25.0	2.3 ± 1.2	2.7 ± 1.2	2.8 ± 0.3	<0.05
prAMEL, 50.0	2.5 ± 1.2	2.8 ± 1.2	3.0 ± 0.5	<0.05
TRAP, 12.5	2.0 ± 1.3	2.6 ± 1.3	2.8 ± 0.4	<0.05
TRAP, 25.0	1.9 ± 2.3	2.6 ± 1.3	2.8 ± 0.4	<0.05
TRAP, 50.0	2.2 ± 1.3	2.5 ± 1.3	2.9 ± 0.6	<0.05
*P* value	0.7	0.04	<0.0001	

The cell index value monitored by the xCELLigence system. Results are from five repeats

*EMP* enamel matrix protein, *EMD* enamel matrix derivative, *prAMEL* porcine recombinant amelogenin, *TRAP* tyrosine-rich amelogenin peptide

### Cell migration

**Fig. 5:**
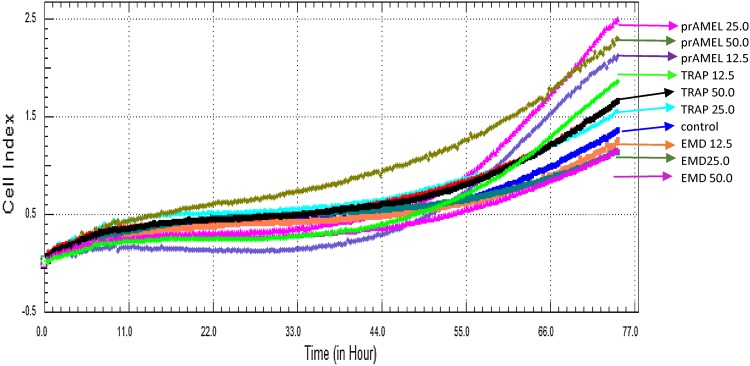
Effect of EMD, prAMEL and TRAP on the rate of migration of HGF-1 cells. The rate of migration was monitored in real-time using the xCELLigence system; representative graph

Cell migration was monitored using RTCA over a period of 72 h after EMD, prAMEL or prTRAP stimulation. Representative graph comparing the rate of CI of HGF cells is shown in Fig. 5. No significant difference in the rate of migration was observed after EMD stimulation, regardless of ligand doses and its stimulation time. RTCA analysis performed after 60- and 72-h incubation showed a significant increase of rate of CI in prAMEL-stimulated cell, for all applied concentrations comparing to both the controls and to the EMD (*P* = 0.0001; Table 2). Moreover, 12.5 µg/mL prTRAP administered for 60 and 72 h caused a significant increase of migration rate in comparison to both the controls and all the applied EMD concentrations (*P* = 0.0001; Table 2).

**Table 2:** Effect of EMPs on rate of migration of HGF-1s cells

EMP added, µg/mL	Cell index value, mean ± standard deviation				
	Time of incubation				
	12-h	24-h	48-h	60-h	72-h
Control	0.26 ± 0.17	0.30 ± 0.20	0.39 ± 0.26	0.56 ± 0.38	0.86 ± 0.60
EMD, 12.5	0.29 ± 0.10	0.35 ± 0.10	0.45 ± 0.13	0.61 ± 0.18	0.88 ± 0.32
EMD, 25.0	0.17 ± 0.11	0.19 ± 0.13	0.24 ± 0.17	0.35 ± 0.27	0.54 ± 0.45
EMD, 50.0	0.26 ± 0.16	0.31 ± 0.19	0.38 ± 0.23	0.47 ± 0.30	0.62 ± 0.43
prAMEL, 12.5	0.28 ± 0.04	0.31 ± 0.02	0.48 ± 0.03	0.90 ± 0.06	1.59 ± 0.13
prAMEL, 25.0	0.35 ± 0.05	0.39 ± 0.01	0.57 ± 0.05	1.24 ± 0.15	1.84 ± 0.26
prAMEL, 50.0	0.19 ± 0.08	0.28 ± 0.12	0.43 ± 0.05	1.01 ± 0.14	1.80 ± 0.22
TRAP, 12.5	0.42 ± 0.06	0.48 ± 0.06	0.66 ± 0.06	1.07 ± 0.15	1.69 ± 0.16
TRAP, 25.0	0.37 ± 0.06	0.41 ± 0.06	0.51 ± 0.05	0.75 ± 0.06	1.09 ± 0.16
TRAP, 50.0	0.48 ± 0.06	0.54 ± 0.06	0.68 ± 0.06	0.95 ± 0.05	1.33 ± 0.10
*P* value	>0.05	>0.05	>0.05	0.0001	0.0001

Cell index values were monitoring using the xCELLigence system. Results are from five repeats

*EMP* enamel matrix protein, *EMD* enamel matrix derivative, *prAMEL* porcine recombinant amelogenin, *TRAP* tyrosine-rich amelogenin peptide

### Cell cycle analysis

**Fig. 6:**
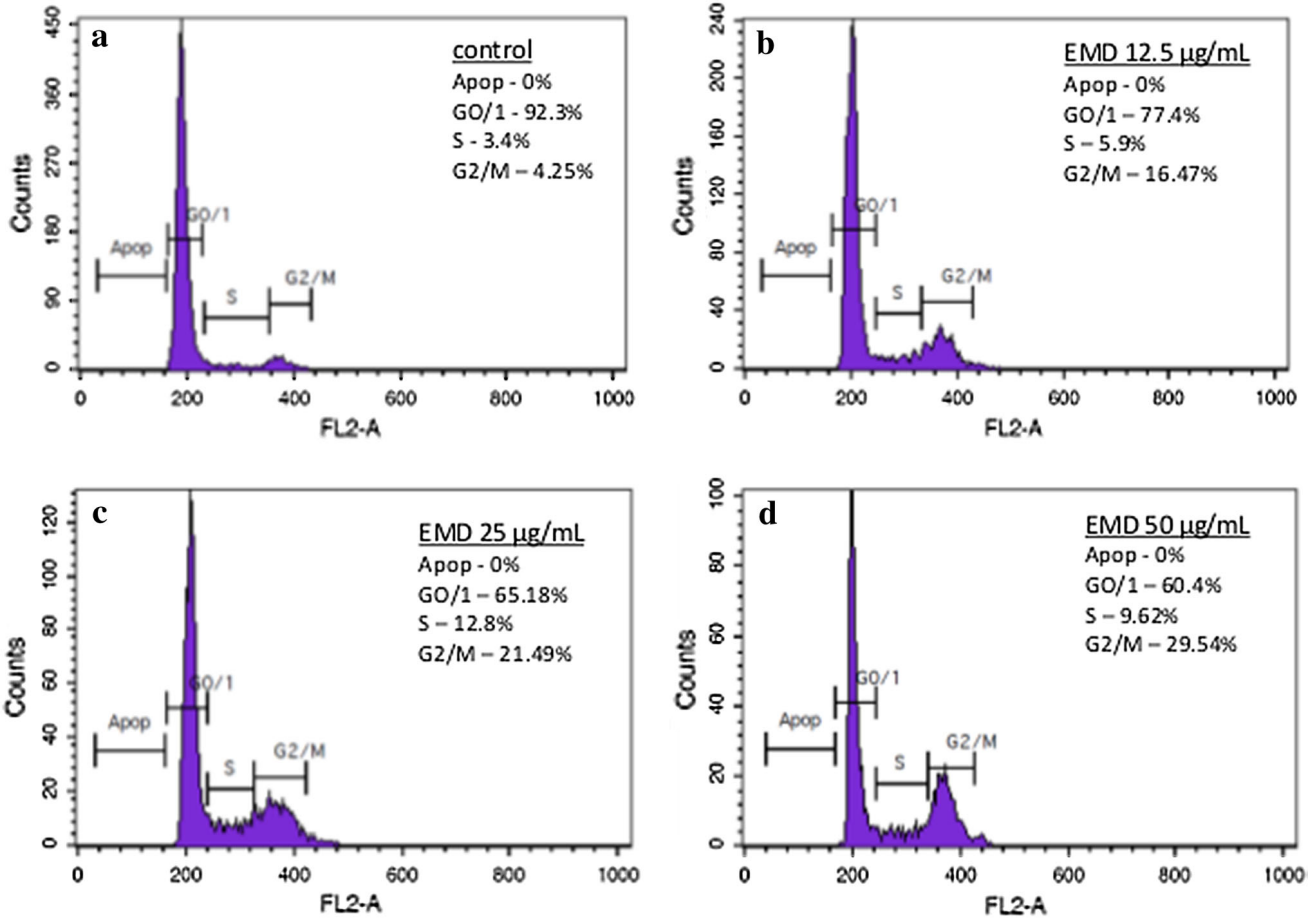
Flow cytometry cell cycle analysis of human gingival fibroblasts in control cells (**a**) and the cells treated with 12.5 (**b**), 25 (**c**) or 50 µg/mL (**d**) EMD, respectively; representative histogram of DNA content

Flow cytometry was used to examine the changes in the cell cycle of the HGF-1 that were either not stimulated or stimulated with EMD, prAMEL or prTRAP. The separation of the cells into apoptotic and the G0/G1, S or G2/M phases was based upon linear fluorescence intensity after staining with PI (Fig. 6).

No apoptosis was observed either in the unstimulated or in the cells stimulated with EMD, prAMEL or prTRAP. In the control group the percentage of cells in the G0/G1, S and G2/M phase was 81.6 ± 7.9, 4.0 ± 2.5 and 11.7 ± 6.6%, respectively.

A significant decrease in the percentage of cells in the G0/G1 phase and a buildup of cells in the S and M phase were observed after EMD and prAMEL stimulation. This process was ligand and concentration-dependent.

**Fig. 7:**
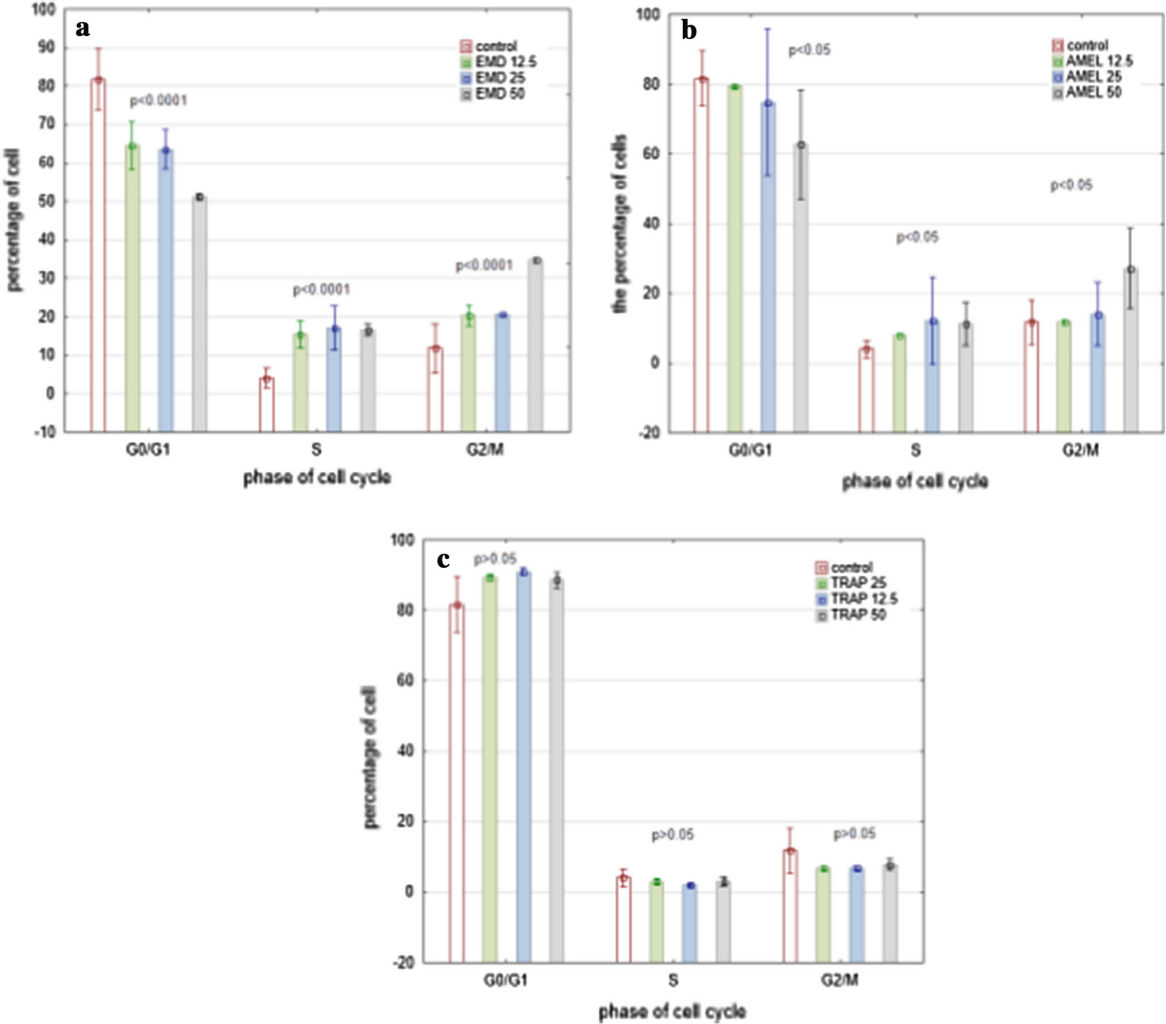
A significant decrease in the percentage of cells in G1 phase and increase in the percentage of cells in S and G2/M cells were observed 48 h after EMD (**a**) or prAMEL (**b**) stimulation. No significant changes in cell cycle were observed after TRAP treatment (**c**)

HGF-1 stimulated with 12.5, 25 or 50 µg/mL EMD had 64.5 ± 6.3, 63.5 ± 5.0 and 51.2 ± 0.7% of cells in the G0/G1 phase, 15.4 ± 3.5, 17.0 ± 5.7 and 16.6 ± 1.4 of cells in the S phase, and 20 ± 2.8%. 20.5 ± 1.1 and 34.7 ± 0.7% of cells in the G2/M phase, respectively (Fig. 7a).

The HGF-1 stimulated with 12.5, 25 or 50 µg/mL prAMEL had 79.5 ± 0.7, 74.8.3 ± 20.9 and 62.7 ± 15.5% of cells in the G0/G1 phase, 7.9 ± 1.1, 12.2 ± 2.8 and 11.2 ± 6.2% of cells in the S phase and 11.8 ± 0.7, 14.1 ± 9.1 and 27.2 ± 11.4% of cells in the G2/M phase, respectively (Fig. 7b).

prTRAP in the concentration of 12.5. 25 or 50 µg/mL did not cause any significant changes in cell percentage concerning each particular cell cycle phase (*P* > 0.05; Fig. 7c).

## Discussion

Influence of enamel matrix proteins (EMP) on periodontal fibroblast is well documented [1]; however, their influence on gingival fibroblast, as well as recombinant amelogenin and its degradation products, is not clear. Moreover, it is difficult to compare the described results, because of different concentrations of stimulants and methods of isolation used in different studies. In vitro study testified that EMD influence on various type cells is not only connected with amelogenin, but also with proteins fractions which independently or in cooperation stimulate the cell reaction reliant of cell type [17, 18]. It was also identified that EMD trigger cell reactions via activation of protein kinase signalling pathway controlled by extracellular kinase-regulating (ERK) or TGF-β receptor [19, 20]. Amelogenin expression was identified in dentin matrix and odontoblasts, in remnants of Hertwig’s root sheath and in PDL cells, bone cartilage cells [21]. It has also been detected in non-mineralizing tissues cells such as brain cells and haematopoetic cells. For this reason recombinant amelogenin is currently investigated as a potential healing or regeneration stimulator in reconstructive medicine [22, 23]. However, until this moment non-specific amelogenin receptor was recognized [24], and only some receptors for different isoforms of amelogenin are suggested. TRAP stimulates angiogenesis [25], but its directed influence on periodontal cells during healing process is uncertain.

We decided to compare biological effect in human gingival fibroblast after EMD stimulation with effects caused by porcine recombinant 21.3-kDa amelogenin and porcine recombinant 5.3-kDa tyrosine-rich amelogenin peptide. The amelogenin construct coded a protein with a mass of 21.3 kDa as well as TRAP construct coded a protein with a mass of 5.3 kDa. However, a GST tag as well as a Histidine tag was added in order to increase the protein solubility.

In the presented study influence of EMD, prAMEL and prTRAP on gingival fibroblast proliferation was monitored in real-time using the xCELLigence RTCA system. The xCELLigence RTCA provides a platform for label-free and operator-independent investigation of the migration, invasion and adhesion proprieties of cells in physiologically relevant conditions. The real-time kinetic data acquisition also allows for a more accurate characterization of short-lived cellular events [27]. The study provided with this technic indicated, that EMD significantly increased HGF proliferation and this reaction is time and dose dependent. 100 μg/mL is the most usual EMD concentration described in literature as the most effective in wound healing (migration and proliferation) promotion. Lyngstadaas et al. [17] even used 500 μg/mL concentration of EMD. However, Bertl et al. [28] observed that 0.1–50 μg/mL of EMD promotes cell migration in the wound healing process and it is inhibited at 100 μg/mL. Also, in other studies it was reported that the EMD at the concentration of 25 μg/mL and lower leads to better results [29–31]. The research on GF cell matrix in response to EMD revealed increased hyaluronan and proteoglycan synthesis. The first reaction was observed in response to 10 μg/mL and proportionally increased with dose up to 150 μg/mL [32]. So, in our study we decided to use 12.5, 25 and 50 μg/mL EMD concentrations. After 12 h of incubation there was no difference in proliferation ratio between all examined ligands and control group. However, after 24 and 48 h of incubation with EMD the difference was statistically significant in all concentration groups. The highest proliferation ratio was observed in 50 μg/mL EMD-stimulated fibroblasts after 48 h of incubation. Our results are similar in the dose-dependent aspect to that presented by other researches [30, 33], but disparate in dynamic processing aspect to results obtained by Kwon et al. [26], who described higher gingival fibroblasts proliferation in EMD 100 μg/mL group compared to control only after 48 h of cell culture. Before this time the proliferation ratio was similar for EMD 25 μg/mL and EMD 100 μg/mL [26]. The author concluded that cell proliferation was not affected by the concentration of the EMD; however, he found cell proliferation of the control group and EMD 25 μg/mL group to be statistically significant [26]. Hoang et al. [34] observed the EMD-stimulated migration and proliferation of HGF starting from 6th day. Also Rincon et al. [33] reported that EMD of 20 μg/mL enhanced wound healing by promoting proliferation and migration of gingival fibroblasts. The difference could have occurred due to incubation time with EMD and applied research technique. Interestingly, in our research with the increase of EMD-stimulated proliferation the same influence on cell migration was not observed. The cell migration and proliferation are often stimulated by the same superior signalling pathway [35, 36]; however, the mechanism responsible for the beginning one of this process is not fully recognized. Cyclin-dependent kinase inhibitors (CDKI) directly modulate both processes and their control by mitogen impulse, may promote migration or proliferation of cells.

Analysis of our data monitored in real-time clearly demonstrated that EMD stimulated the proliferation of HGF. These observations were confirmed by cell cycle analysis, which showed a significant decrease in the percentage of cells in the G0/G1 phase and a buildup of cells in the S and M phase after EMD. This process was ligand and concentration-dependent. Zeldich et al. [20] also observed that EMD synergistically induced completion of the cell cycle, resulting in increased cell number. They suggested that mitogenic response to EMD depended on extracellular-regulated kinase (ERK) activation.

The ability of a 25-kDa recombinant amelogenin to affect cell proliferation, adhesion and migration is likely to be dependent on the cell type. Our observation indicated amelogenin as the stimulant of gingival fibroblast proliferation and migration. The proliferation was dose-dependent. After 24 h of cell culture the prAMEL-stimulated proliferation ratio was significant for all concentrations used, and higher comparing to the control group. Interestingly, the 2nd day after incubation is a natural moment of proliferation growth for gingival fibroblasts in an in vitro wound model [37]. The higher proliferation ratio was observed until 48 h of cell incubation and this difference was statistically significant for prAMEL 50 μg/mL. Its mitogenic response was indicated by the analysis of the cell cycle of the cells stimulated with AMEL. These observations are consistent with current literature. Van der Pauw et al. [31] also described the increase of HGF proliferation after the addition of porcine recombinant amelogenin, as well as Grayson et al. [38] observed increase of the number of dermal fibroblast after contact with amelogenin starting on the 7th day. We also found that prAMEL significantly increased HGF migration. After stimulation for all amelogenin concentrations, the results were statistically significant and higher than that obtained after TRAP and EMD supplementation. The increased HGF migration was most apparent on the 3rd day after incubation. Lallier et al. [40] using tissue-culture flask models even showed that the rate of gingival fibroblast motility significantly increased after 7 days in culture. Because recombinant amelogenin significantly increased GF adhesion during the first 60 min of incubation [32], it may prove the observation that cell motility rates did not correlate with cell proliferation and inversely correlate with cell attachment [40]. However, Li et al. [14] found a significant decrease in the rate of migration of GF and gingival epithelial cell (GEC) in response to porcine recombinant amelogenin treatment, which is only similar to the results we obtained with reference to GEC [39]. However, Li et al. [14] also suggest the beginning of changes after first 6 h of incubation, because in first few hours GF did not have the ability to migrate.

In the literature, there are only few publications connected with TRAP-induced healing of periodontal tissue. It is suggested that the synthetic fragment of the amelogenin peptide (TRAP) (45 amino acids N-terminal) did not promote wound healing. Stout et al. [41] did not find biologic activity for TRAP, and Amin et al. [42] found its suppressive effect on periodontal ligament and alveolar bone. TRAP seems not to participate in stimulation of both gingival epithelial cells and fibroblasts. It is even suggested that this fraction acts by a receptor-mediated endocytosis mechanism rather than by a precipitation-related process, typical for EMD and recombinant mouse amelogenin [43]. In the present study, no significant difference in proliferation ratio and cell cycle was observed after prTRAP stimulation. Nevertheless, in our observation prTRAP has had the ability for healing stimulation by positive effect especially on migration of gingival fibroblasts. TRAP in all concentrations stimulated HGF migration and in 12.5 TRAP this difference in comparison with control group was statistically significant. Due to a lack of published data on the effects of prTRAP on the migration of cultured human gingival fibroblasts, it is difficult to place these results in context with other studies. However, Jonke et al. [26] showed that both TRAP isolated from EMD and chemically synthesized TRAP, stimulated endothelial cell migration in microchemotaxis chamber and the migration was significantly higher than that of EMD. This effect was observed when 50 μg/ml TRAP was used. Changes in the proliferation ratio after prTRAP stimulation, was considerably smaller than by the EMD and amelogenin groups. Moreover, differences were significant only for prTRAP 50 μg/mL concentrations and were detected after 60 h of cell culture only. The same observations were made by Jonke et al. [26], who described TRAP in concentration 100 μg/mL having an inhibiting effect on endothelial cell proliferation. The observed differences in cell behaviour, particularly after TRAP stimulation seem to be important when TRAP was isolated from EMD, chemically synthesized and recombinant TRAP is used. In the present study, we decided to use both recombinant amelogenin and TRAP; moreover, there were porcine AMEL and TRAP. Those proteins are conserved in human and other mammals [44]; however, EMD contains porcine proteins, so we decided to minimize any differences related to these.

## Conclusion

Primary function of the fibroblast is to deposit and remodel the extracellular matrix (ECM). Heterogeneity of fibroblast is connected mainly with ECM [45, 46]. Because EMD has the ability to bind to extracellular matrix proteins and regulate their adhesive properties, it is postulated that EMD can control cell-to-cell relationship [47]. EMD relevantly stimulate gingival fibroblasts proliferation. This effect was significantly higher than the one observed after recombinant amelogenin stimulation which suggests that during the healing process the proliferation is the predominant cell reaction in the early stage after enamel matrix proteins treatment and it is the result of synergistic cellular function of other components [48] or new factors present in the EMP. Amelogenin alone sustains gingival fibroblast proliferation started by EMD, but essentially stimulates fibroblast migration. It may be of importance during the healing and regeneration process when the local fibroblasts are damaged or injured and the new potential cells are needed. Involvement of porcine recombinant TRAP in gingival proliferation is certain which is in agreement with cementoblasts reaction after TRAP stimulation [49]. However, prTRAP alone or as a part of proteolytically processed amelogenin, might promote healing as a stimulant of gingival fibroblast migration.
